# Supplementary material for: LightCue: An Innovative Far-Red Light Emitter for Locally Modifying the Spectral Cue in Outdoor Conditions with Global Consequences on Plant Architecture
Source: Plants (Basel). 2021 Nov 17;10(11):2483. doi: 10.3390/plants10112483 (PMC8625856; doi:10.3390/plants10112483)
Supplement: Supplementary file 1 [file plants-10-02483-s001.zip › plants-1456553-supplementary.pdf]

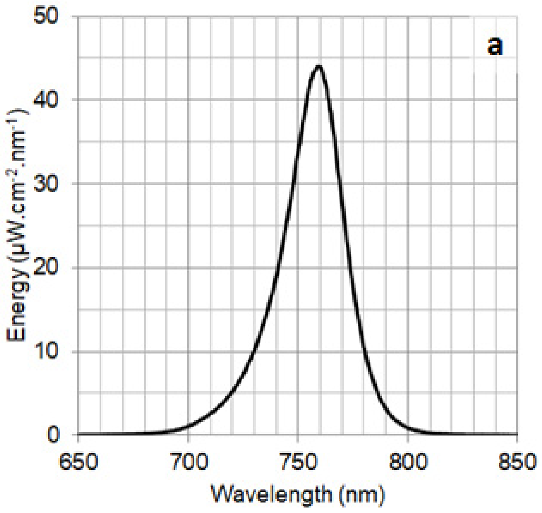

Supplementary Figure S1: (**a**) Irradiance spectrum of Far-Red LED measured at 2.5 cm from one LED using a spectroradiometer. (**b**) FR Photon Flux Density (PFD) response as a function of the distance to one LED (µmol.m-2.s-1) measured with a spectroradiometer.

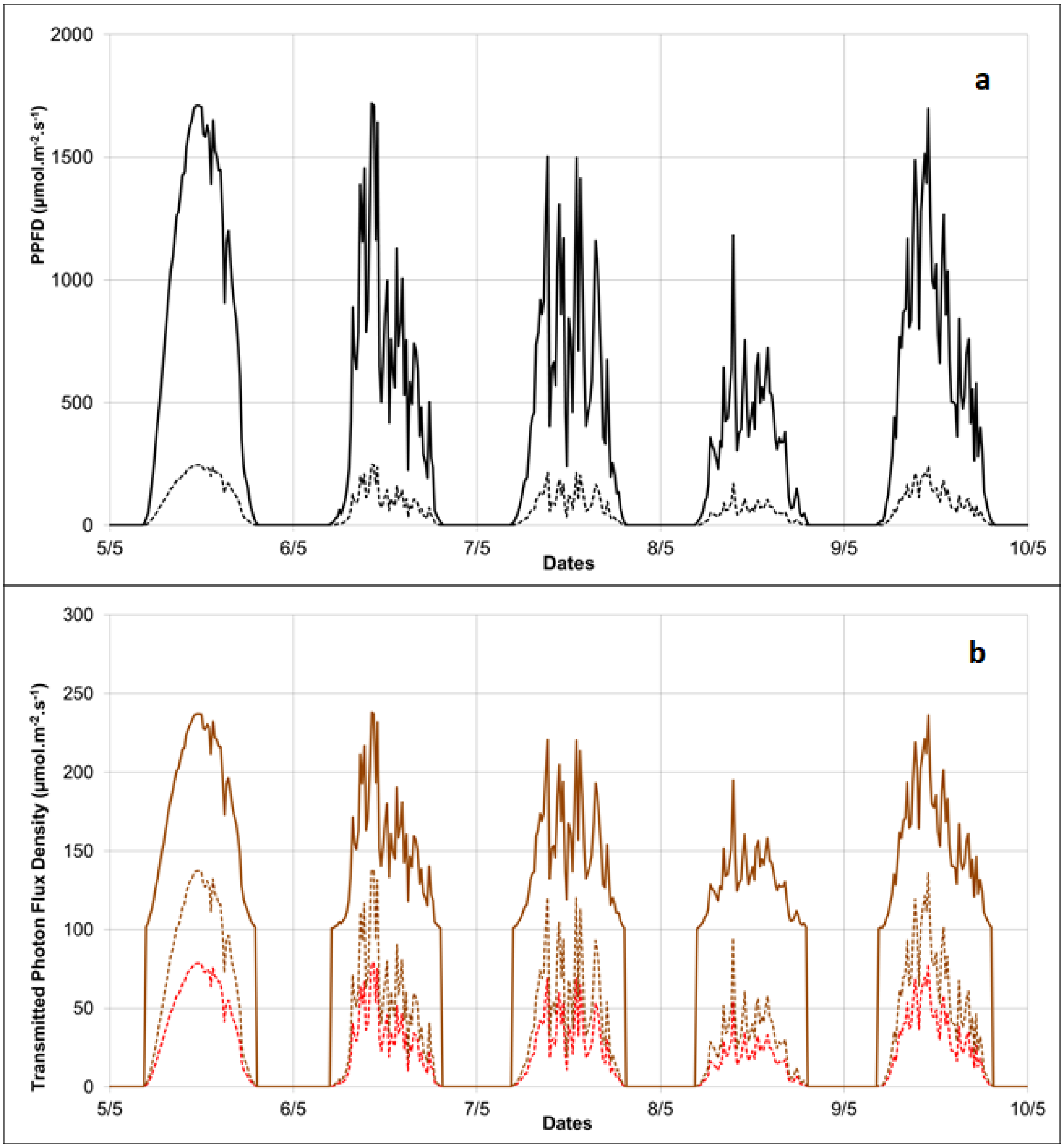

Supplementary Figure S2. (**a**) incident PPFD (solid line) and transmitted PPFD (dashed line). (**b**) average transmitted radiation at the base of the plants equipped with LightCue, transmitted Red (red dashed line, Rt 600-700 nm); transmitted Far-red (brown dashed line, FRt 700-800 nm) and FRt + LightCue PFD (brown line).
